# CD23 sustains NKT17 cell homeostasis via IL-7Rα-mTORC2 signaling

**DOI:** 10.1038/s41419-026-09060-x

**Published:** 2026-06-30

**Authors:** Linlin Niu, Bingqian Tan, Jingting Xu, Guangwei Zhang, Bo Wang, Zuochen Du, Di Yang, Chengxi Zhuang, Xiaoyu Du, Yao Zhao

**Affiliations:** 1 National Clinical Research Center for Children and Adolescents’ Health and Diseases, Ministry of Education Key Laboratory of Child Development and Disorders, Chongqing Key Laboratory of Pediatric Metabolism and Inflammatory Diseases, Pediatric Research Institute, Children’s Hospital of Chongqing Medical University, Chongqing, China; 2 Department of Hepatobiliary Surgery, Children’s Hospital of Chongqing Medical University, Chongqing, China; 3 Chongqing Key Laboratory of Child Infection and Immunity, Children’s Hospital of Chongqing Medical University, Chongqing, China; 4 Collaborative Innovation Center for Tissue Injury Repair and Regenerative Medicine of Zunyi Medical University, Zunyi, China

**Keywords:** Innate immunity, Innate lymphoid cells

## Abstract

Invariant natural killer T (iNKT) cells serve as a crucial bridge between innate and adaptive immunity, yet the molecular mechanisms that sustain their subset homeostasis remain incompletely defined. Here, we identify CD23 (FcεRII) as a previously unrecognized intrinsic regulator of NKT17 cell homeostasis. CD23 deficiency selectively diminished NKT17 frequency and IL-17 production in the thymus and spleen, while leaving NKT1 and NKT2 subsets largely unaffected. Loss of CD23 led to decreased IL-7Rα expression and impaired mTORC2-dependent phosphorylation of AKT (Ser473), accompanied by reduced Bcl-2 expression and increased apoptosis of NKT17 cells. Administration of exogenous IL-7 restored NKT17 abundance and IL-17 secretion in CD23-deficient mice without normalizing PLZF or RORγt expression, indicating that CD23 primarily supports metabolic and survival signaling rather than transcriptional programming. Collectively, these findings define CD23 as an upstream checkpoint of the IL-7Rα-mTORC2 axis that preserves NKT17 viability. By delineating a previously unrecognized link between cytokine and metabolic pathways, this study expands the molecular framework governing iNKT cell development and subset maintenance.

**Figure:**
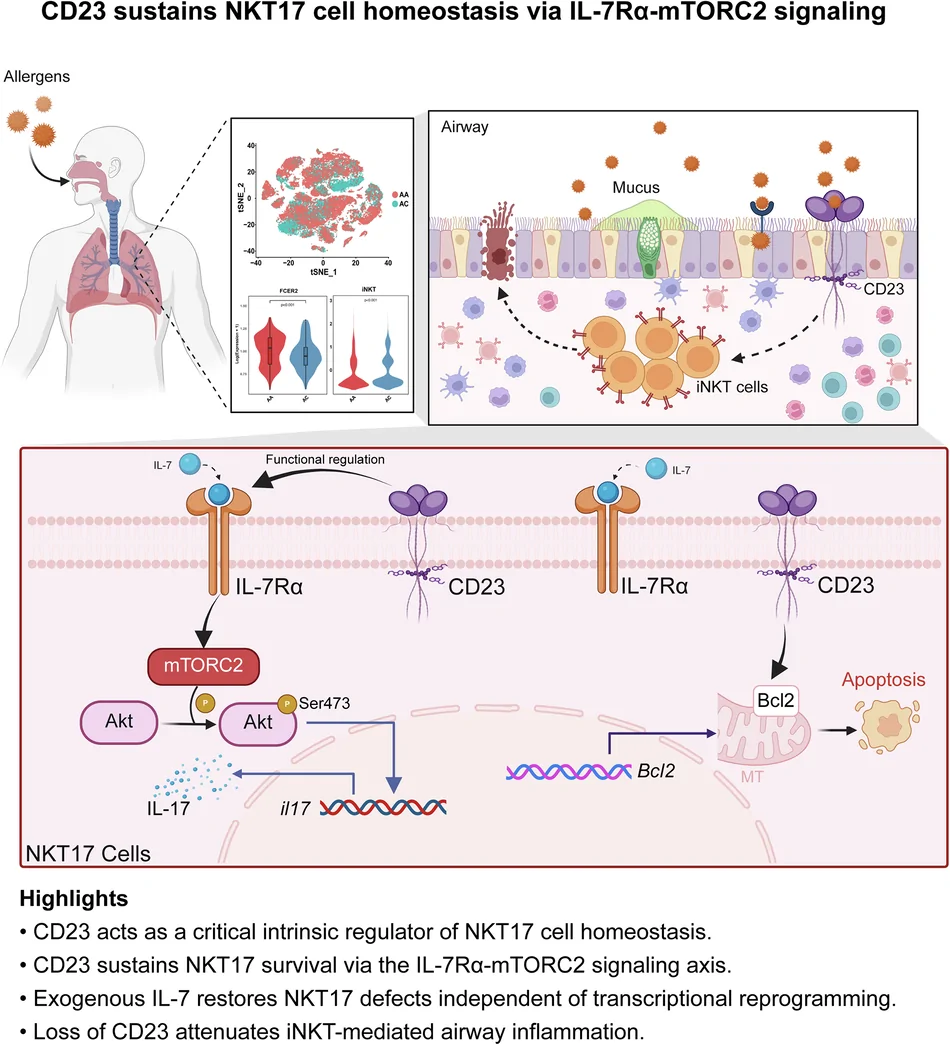

## Introduction

Invariant natural killer T (iNKT) cells constitute a specialized subset of T lymphocytes that recognize lipid antigens presented by the monomorphic MHC class I-like molecule CD1d. These cells are uniquely characterized by their activation via the prototypical glycolipid antigen α-galactosylceramide (α-GalCer) through a semi-invariant T cell receptor (TCR). This TCR comprises an invariant α-chain (Vα14-Jα18 in mice and Vα24-Jα18 in humans) paired with a restricted repertoire of β-chains (predominantly Vβ8, Vβ7, and Vβ2 in mice, and Vβ11 in humans) [1, 2]. The developmental trajectory of iNKT cells is governed by a network of transcription factors, with promyelocytic leukemia zinc finger (PLZF) serving as a master regulator. PLZF is indispensable for orchestrating both early thymic development and functional polarization of iNKT cells [3]. Recent advances in lineage classification have further delineated these cells into three distinct effector subsets—NKT1, NKT2, and NKT17—defined by their signature transcription factors (T-bet, GATA3, and RORγt, respectively) and cytokine profiles [4, 5]. Notably, iNKT cells exhibit rapid and robust cytokine secretion, releasing mediators such as IL-4, IFN-γ, IL-17, and IL-13 within minutes of activation. This prompt response enables them to act as pivotal intermediaries bridging innate and adaptive immunity [6]. Accumulating evidence underscores their critical immunomodulatory roles in diverse pathological contexts, particularly in respiratory diseases and chronic inflammatory disorders [7, 8]. Among iNKT subsets, NKT17 cells have garnered significant attention due to their predominant production of IL-17. While this cytokine is essential for maintaining mucosal barrier integrity and host defense, its dysregulated overexpression has been mechanistically linked to pathogenic processes in allergic asthma. Specifically, IL-17 contributes to airway hyperresponsiveness (AHR), neutrophilic infiltration, and structural remodeling of bronchial tissues, as demonstrated in both murine models and human clinical studies [9–11].

CD23 (FcεRII), a type II transmembrane glycoprotein belonging to the C-type lectin family, serves as a critical modulator of immunoglobulin E (IgE)-mediated immune regulation. It plays a significant role in the regulation of immune responses, particularly in modulating allergic reactions and inflammatory processes [12]. This low-affinity IgE receptor is predominantly expressed on B lymphocytes, though emerging evidence confirms its co-expression on activated T cell subsets, particularly under inflammatory conditions [13]. Notably, murine models with targeted deletion of the *Fcer2* gene (CD23-deficient mice) have provided pivotal insights. Although these animals exhibit unperturbed development of both T and B lymphocyte lineages [14], they demonstrate marked dysregulation in IgE production [15]. Furthermore, CD23 deficiency profoundly alters pathological manifestations in ovalbumin-induced asthma models, characterized by attenuated eosinophilic airway inflammation yet paradoxically enhanced airway hyperresponsiveness (AHR) [16]. However, the regulatory mechanisms through which CD23 modulates the functional dynamics of invariant natural killer T (iNKT) cells within the innate immune system remain incompletely elucidated.

The mechanistic target of rapamycin (mTOR) is a serine/threonine kinase of the phosphoinositide 3-kinase-related kinase (PIKK) family, which acts as a master regulator of cellular metabolism, survival, and proliferation by dynamically balancing energy production and consumption [17, 18]. This kinase forms two distinct complexes: mTORC1 (containing the scaffolding protein Raptor) and mTORC2 (defined by its core subunit Rictor), which mediate divergent biological functions [19]. In recent years, the mTOR signaling pathway—especially the mTORC2 complex—has been identified as playing a pivotal role in the development, differentiation, and functional regulation of invariant natural killer T (iNKT) cells and NKT17 cells [20]. Specifically, mTORC2 influences the differentiation of NKT17 cells and the secretion of interleukin-17 (IL-17) through the phosphorylation of AKT at serine 473 (Ser473) [21]. However, the upstream regulatory mechanisms that control mTORC2 activity in this context remain incompletely understood. Furthermore, the IL-7/IL-7R signaling axis is essential for the survival and homeostatic maintenance of conventional T cells as well as iNKT cells [22, 23]. Intriguingly, although CD23 (FcεRII) is established as a key mediator of IgE-dependent antigen presentation and Th2 polarization in allergic inflammation, its potential involvement in iNKT cell metabolic regulation remains an uncharted territory.

Clinically, scRNA-seq profiling of human allergic asthmatic bronchial brushings reveals a concurrent upregulation of *FCER2* (CD23) and altered iNKT cell signatures. Driven by this translational rationale, we identify CD23 as a critical regulator of iNKT cell development and subset maintenance. CD23 deficiency disrupted iNKT maturation in the thymus and spleen, leading to a selective loss of NKT17 cells and reduced IL-17 production, while NKT1 and NKT2 subsets remained largely intact. CD23 sustains NKT17 survival by maintaining IL-7Rα expression and activating mTORC2-AKT (Ser473) signaling. Loss of CD23 reduces Bcl-2 expression and increases NKT17 apoptosis. Exogenous IL-7 restored NKT17 numbers and IL-17 secretion in CD23-deficient mice, underscoring that this pathway primarily regulates cell survival rather than lineage programming controlled by RORγt and PLZF, and highlighting the essential role of IL-7Rα-mTORC2 signaling in iNKT homeostasis. Together, these findings position CD23 as an upstream checkpoint linking IL-7Rα expression to mTORC2 activation, thereby preserving NKT17 viability and refining the molecular framework of iNKT cell development.

## Results

### Single-cell transcriptome analysis reveals immune cell lineage alterations in allergic asthma

To investigate immune cell composition and transcriptional profiles in the bronchial mucosa of allergic individuals, we analyzed single-cell transcriptomic data from bronchial brush samples in a human allergic asthma exacerbation model (Alladina et al., 2023; GSE193816). Following quality control, dimensionality reduction, clustering, and cell type annotation, downstream analyses were conducted using public databases, including immune cell signature scoring, subpopulation proportion assessment, and key gene expression profiling [24]. t-SNE analysis (Supplementary Fig. S1A) revealed substantial overlap in overall immune cell distribution between allergic asthmatics (AA) and allergic controls (AC). Clustering and annotation identified multiple immune cell subpopulations, including CD4^+^ and CD8^+^ T cell subsets, invariant natural killer T (iNKT) cells, and mucosal-associated invariant T (MAIT) cells (Supplementary Fig. S1B). Quantitative analysis showed inter-individual variability, yet specific T cell subsets exhibited pronounced differential trends between AA and AC groups (Supplementary Fig. S1C). Comparison of immune cell signature scores revealed significant differences in multiple subpopulations. Specifically, AA samples exhibited altered signature scores in CD4^+^ naive T cells, CD8^+^ T cells, iNKT cells, regulatory T cells (Tregs), naive and memory B cells, activated natural killer (NK) cells, and M1/M2 macrophages, whereas CD4^+^ memory T cells were unchanged (*p* = 0.347) (Supplementary Fig. S1D). Within the B cell compartment, CD81 forms a functional complex with CD19, CD21, and CD225, enhancing B cell receptor (BCR) signaling and contributing to T cell activation and antiviral responses [25, 26]. Gene expression analysis revealed that *CD81* was significantly downregulated in AA samples, while *CR2* (CD21) and *CD19* expression remained unchanged (Supplementary Fig. S1E). Examination of IgE receptor-related genes showed decreased *FCER1G* (encoding the FcεRIγ chain), markedly increased *FCER2* (encoding CD23), and unchanged *FCER1A* (encoding the FcεRIα chain) expression in AA samples compared with AC (Supplementary Fig. S1F). Collectively, these results indicate substantial remodeling of immune cell composition and functional states in the bronchial mucosa of allergic asthmatics, with notable dysregulation of molecules involved in B cell activation and IgE receptor signaling, highlighting their potential roles in asthma-associated immune dysregulation.

### Loss of CD23 impairs the development of iNKT cells

**Fig. 1:**
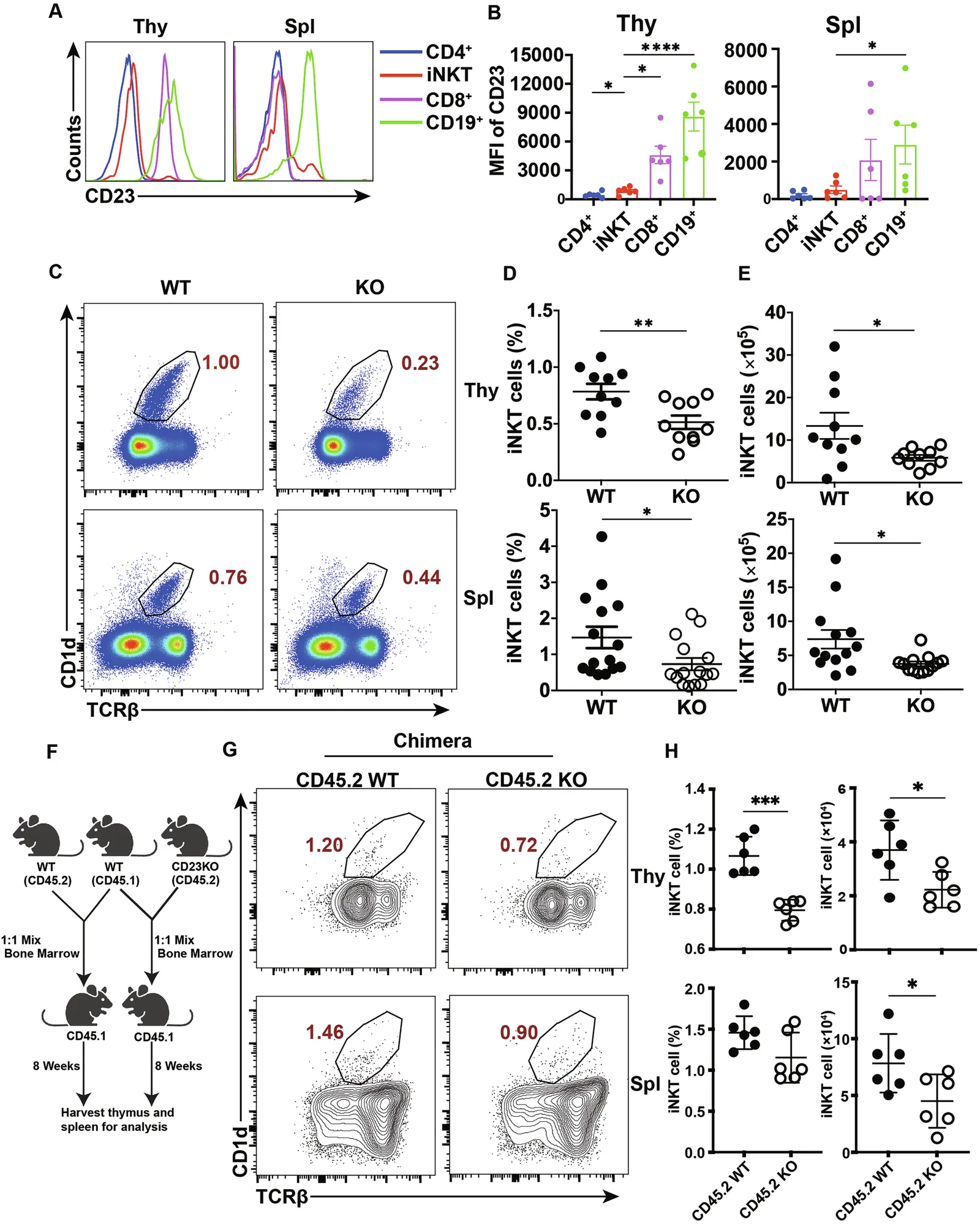
Loss of CD23 impairs the development of iNKT cells. **A**, **B** The surface expression of CD23 on CD4^+^ T cells, iNKT cells, CD8^+^ T cells, and CD19^+^ B cells in thymus and spleen from BALB/c mice (*n* = 6). **C** Flow cytometry analysis of iNKT (CD1dTet^+^TCRβ^+^) cells from the thymus and spleen in WT and CD23 KO mice. Representative dot plots showing 7-AAD^-^ cells. **D**, **E** Percentages (as gated in **C**) and total numbers of iNKT cells in the thymus (*n* = 10) and spleen (*n* = 15) from WT and CD23 KO mice. **F** Experimental scheme. Bone marrow cells from CD45.1, CD45.2 (WT) and CD45.2 (CD23 KO) were isolated and mixed at a ratio of 1:1 (CD45.1: CD45.2 (WT) or CD45.1: CD45.2 (CD23 KO)) and injected (i.v.) into CD45.1 recipient mice, eight weeks after reconstitution, the thymus and spleen were harvested for analysis. **G** Flow cytometry analysis of iNKT (CD1dTet^+^TCRβ^+^) cells from thymus and spleen in CD45.2 WT and CD45.2 KO mice. **H** Percentages (as gated in **G**) and total numbers of iNKT cells in the thymus (*n* = 6) and spleen (*n* = 6) from CD45.2 WT and CD45.2 KO mice. Data are presented as mean ± SEM. Statistical significance was assessed using one-way ANOVA or a two-tailed unpaired Student’s *t* test. *p < 0.05, **p < 0.01, ***p < 0.001, ****p < 0.0001.

Given that single-cell transcriptomic analysis revealed significantly upregulated CD23 (*FCER2*) expression in the bronchial mucosa of allergic asthmatic (AA) patients (Supplementary Fig. S1F), alongside impaired functional signature scores of iNKT cells in the AA group (Supplementary Fig. S1D), we further investigated whether CD23 directly regulates the development and function of iNKT cells. To this end, using a CD23-deficient mouse model, we systematically evaluated the cell-intrinsic role of CD23 in iNKT cell development within the thymus and spleen. We first analyzed the surface expression of CD23 on iNKT cells. We observed that CD23 was expressed on CD4^+^ T cells, CD8^+^ T cells, iNKT cells and B cells in BALB/c mice (Fig. 1A). In thymocytes, the expression of CD23 on iNKT cells was higher than that on CD4^+^ T cells, whereas in splenocytes, there was no difference in the expression of CD23 between iNKT cells and CD4^+^ T cells (Fig. 1A, B). To determine the role of CD23 in the development of iNKT cells, we examined the frequency and number of iNKT cells in the thymus and spleen in CD23-deficient mice. Compared with WT mice, deleting CD23 significantly inhibited iNKT cell development, as indicated by reduced iNKT cell frequency and numbers in the thymus and spleen (Fig. 1C–E). To determine whether CD23-dependent regulation of iNKT cell development is cell-intrinsic, we generated mixed bone marrow chimeric mice reconstituted with CD45.1 WT bone marrow cells and either CD45.2 WT or CD23 KO bone marrow cells at a 1:1 ratio (Fig. 1F). We found that the percentage of CD45.2 CD23 KO iNKT cells was decreased in the thymus (Fig. 1G, H). Mixed bone marrow chimeric mice revealed a reduction in total iNKT cells of the thymus and spleen in CD45.2 CD23 KO mice (Fig. 1H). Altogether, these data reveal CD23 as a cell-intrinsic regulator for iNKT cell development and maturation.

### CD23 promotes differentiation of NKT17 cells

**Fig. 2:**
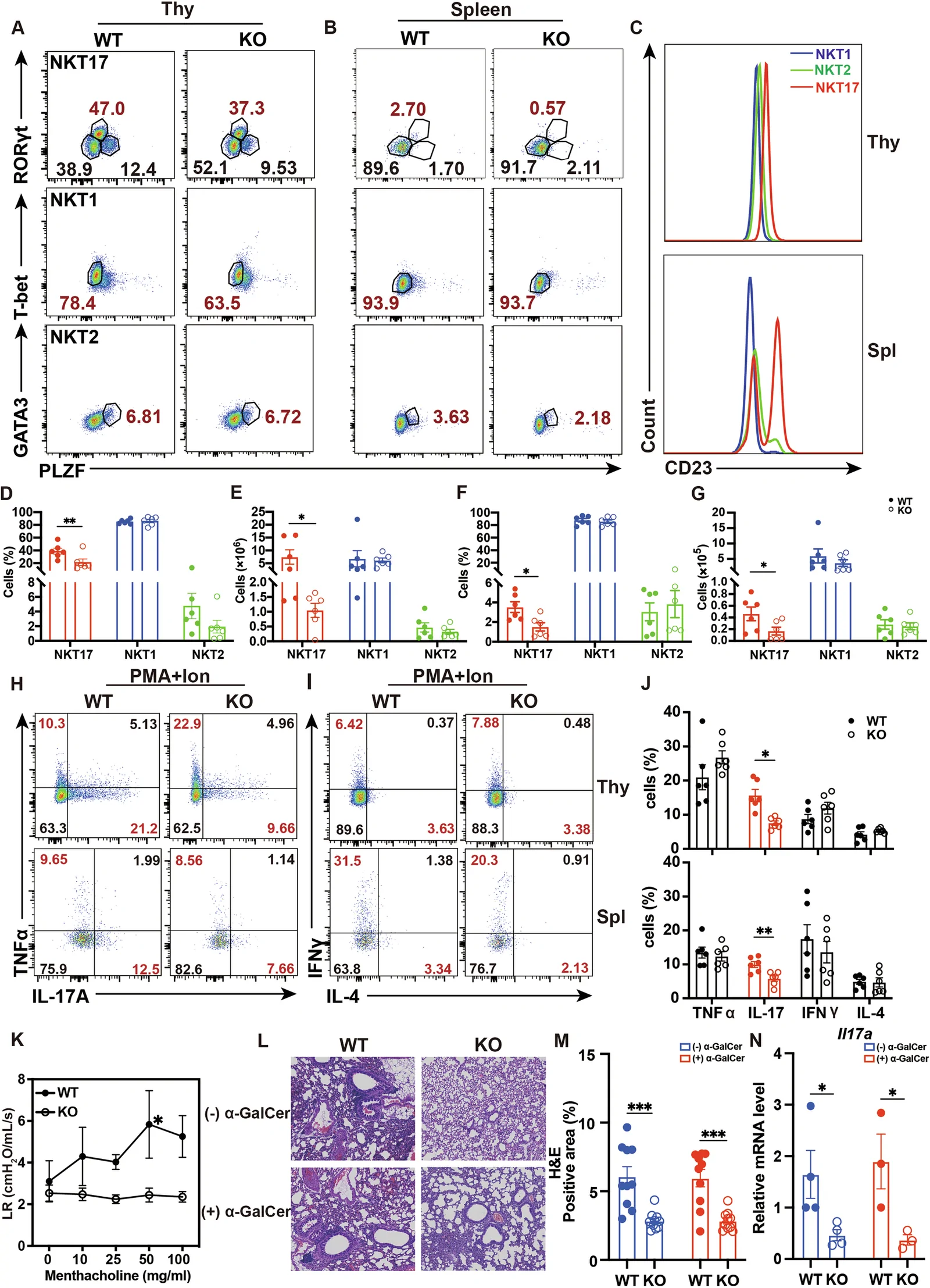
CD23 promotes differentiation of NKT17 cells. **A**, **B** Gating strategy for identifying NKT1, NKT2, and NKT17 subsets from the thymus and spleen of WT and CD23 KO mice. **C** The surface expression of CD23 on NKT1, NKT2, and NKT17 subsets in the thymus and spleen of BALB/c mice. **D**–**G** Percentages and total numbers of NKT1, NKT2, and NKT17 cells in the thymus (*n* = 6) and spleen (*n* = 6) from WT and CD23 KO mice. **H**, **I** Gating strategy for cytokine production by iNKT cells (gated on CD1d-PBS57^+^TCRβ^+^ cells) from the thymus and spleen following 5 h of stimulation with PMA and ionomycin. **J** Production of IFNγ, IL-4, IL-17, and TNFα by iNKT cells from the thymus and spleen in WT and CD23 KO mice (*n* = 6 per group). **K** Airway resistance to increasing doses of aerosolized methacholine in WT and CD23 KO mice 24 h after intranasal (i.n.) challenge with α-GalCer (2 μg in 50 μL PBS). LR, lung resistance. **L** CD23 deficiency alleviates lung inflammation. Representative H&E staining of lung sections showing decreased interstitial infiltration in CD23 KO mice compared to WT controls (original magnification, ×200). **M** Quantification of the interstitial infiltration area shown in (**L**). Positive staining areas were measured using ImageJ software. **N** Relative *Il17a* mRNA expression in the lungs of WT and CD23 KO mice 2 h after vehicle or α-GalCer treatment. Data are presented as mean ± SEM. Statistical significance was determined by two-way ANOVA. *p < 0.05, **p < 0.01, ***p < 0.001.

It is well known that iNKT cells differentiate into NKT1, NKT2, and NKT17 subsets, defined by the expression of PLZF coupled with other signature transcription factors [2]. To reveal the influence of CD23 on iNKT cell differentiation, we investigated NKT1, NKT2, and NKT17 cells by PLZF staining as well as T-bet, GATA3 and RORγt. In BALB/c mice, CD23 expression was markedly enriched in NKT17 cells, whereas only low levels were detected in NKT1 and NKT2 cells within both the thymus and spleen (Fig. 2A–C). In the thymus of CD23-deficient mice, the proportion of NKT17 cells was significantly reduced, whereas the frequencies of NKT1 and NKT2 cells remained unchanged (Fig. 2D), highlighting a selective role of CD23 in iNKT subset development. Consistently, analysis of absolute cell numbers revealed a marked reduction of NKT17 cells, while NKT1 and NKT2 cell numbers were unaffected (Fig. 2E). Similar reductions in both the proportion and absolute number of NKT17 cells were observed in the spleen of CD23-deficient mice (Fig. 2F, G). We further found that the frequency and absolute number of NKT17 cells were reduced in the thymus of CD45.2 CD23-deficient mice, whereas NKT1 and NKT2 cells were unaffected (Supplementary Fig. S2A, B). In contrast, no significant differences in the frequencies or numbers of NKT1, NKT2, or NKT17 cells were observed in the spleens of CD45.2 CD23-deficient and CD45.2 WT mice (Supplementary Fig. S2A, C). Taken together, these results indicate that CD23 is required for NKT17 cell development in the thymus but is dispensable for the development of NKT1 and NKT2 subsets.

To investigate the role of CD23 in regulating iNKT cell effector functions, we first stimulated thymic and splenic iNKT cells with PMA and ionomycin. The production of IL-4, TNFα, and IFNγ was comparable between WT and CD23-deficient mice; however, IL-17 production was markedly reduced in CD23 KO iNKT cells in both the thymus and spleen (Fig. 2H–J). Consistently, when thymic and splenic cells were stimulated in vitro with the lipid antigen α-GalCer for three days, CD23-deficient iNKT cells produced significantly less IL-17 than WT cells in the thymus, whereas IL-4, TNFα, and IFNγ production remained comparable (Supplementary Fig. S2D–F). Furthermore, intraperitoneal administration of α-GalCer followed by intracellular cytokine staining demonstrated that CD23 deficiency selectively impaired IL-17 production in thymic iNKT cells, while IFNγ, TNFα, and IL-4 expression was unaffected (Supplementary Fig. S2G–I). In addition, analysis of CD45.2 WT and CD45.2 CD23 KO mice confirmed that IL-17 production was reduced in thymic iNKT cells from CD23-deficient mice (Supplementary Fig. S2J–L). Collectively, these findings demonstrate that CD23 is essential for IL-17 production in iNKT cells.

To evaluate the impact of CD23 deficiency on iNKT cell function in vivo, we examined α-GalCer–induced changes in airway homeostasis, focusing on airway hyperresponsiveness (AHR). IL-17 produced by iNKT cells is known to promote bronchial smooth muscle contraction and neutrophil infiltration, thereby enhancing airway resistance [9, 10, 27]. Twenty-four hours after α-GalCer administration, CD23-deficient mice exhibited significantly lower AHR compared with WT controls (Fig. 2K). Consistently, histological analysis revealed markedly reduced inflammatory cell infiltration in the pulmonary interstitial tissue of CD23 KO mice following α-GalCer challenge (Fig. 2L, M), which was accompanied by a downregulation of *Il17a* mRNA expression (Fig. 2N). Collectively, these findings demonstrate that CD23 deficiency attenuates IL-17 production by iNKT cells in vivo, leading to reduced airway hyperresponsiveness and diminished pulmonary inflammation.

### CD23 disparately controls survival of iNKT subsets in the thymus

**Fig. 3:**
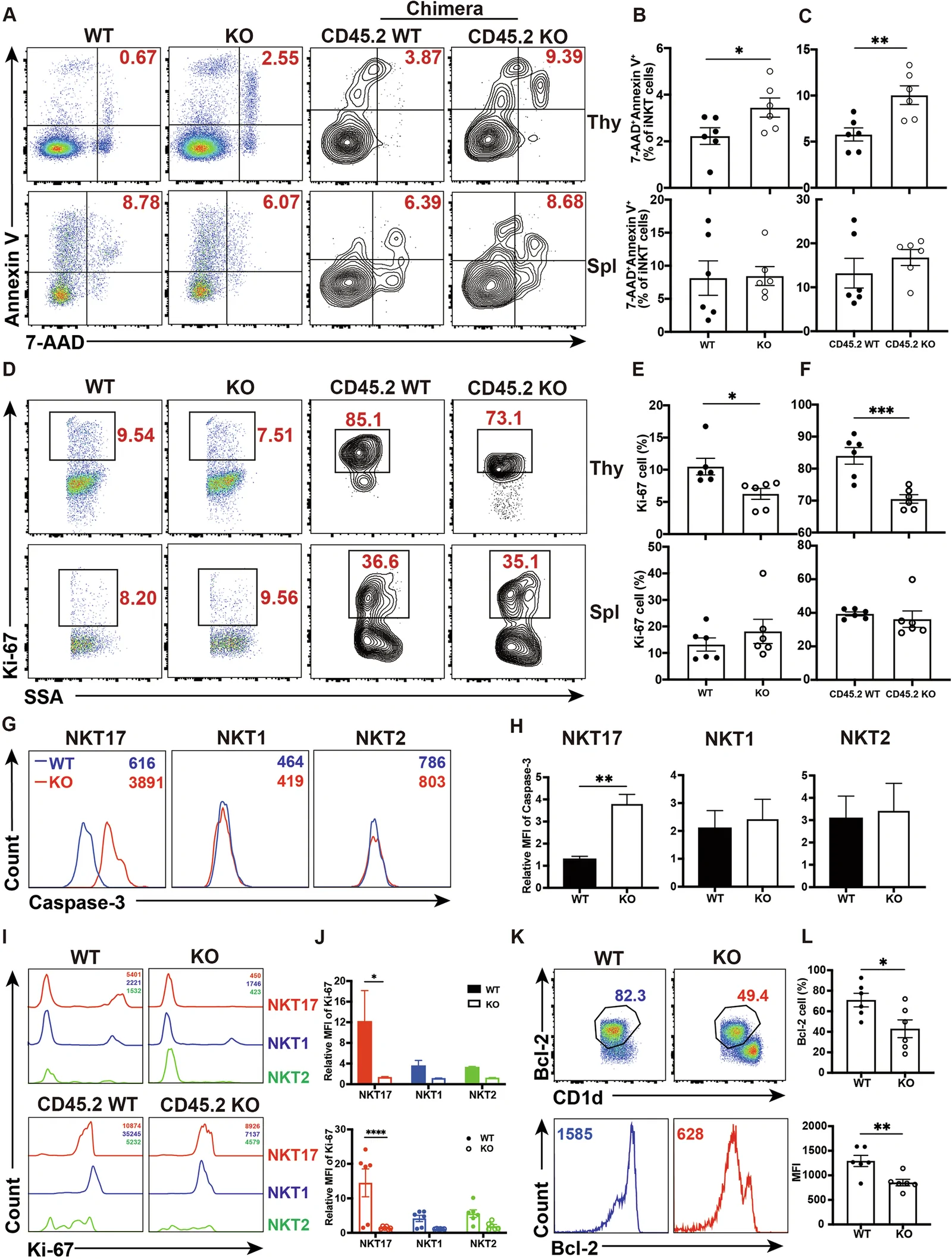
CD23 disparately controls survival of iNKT subsets in the thymus. **A** Gating strategy for assessing apoptosis in iNKT cells from WT, CD23 KO, CD45.2 WT and CD45.2 CD23 KO mice using 7-AAD and Annexin V staining. **B**, **C** Quantification of 7-AAD and Annexin V positive iNKT cells in the thymus (*n* = 6) and spleen (*n* = 6) from WT, CD23 KO, CD45.2 WT, and CD45.2 KO mice. **D** Representative flow cytometric analysis of Ki-67 expression in thymic and splenic iNKT cells from WT, CD23 KO, and bone marrow chimeric mice. **E**, **F** Percentages of Ki-67-positive iNKT cells in the thymus (*n* = 6) and spleen (*n* = 6) from WT, CD23 KO, CD45.2 WT, and CD45.2 KO mice. **G** Representative histograms of Caspase-3 staining in thymic NKT1, NKT2, and NKT17 subsets from WT and CD23 KO mice. **H** Quantification of Caspase-3 mean fluorescence intensity (MFI) showing significantly increased apoptosis in the NKT17 subset of CD23 KO mice. **I**, **J** Analysis of proliferation in different iNKT subsets from WT, KO, CD45.2 WT, and CD45.2 KO mice using Ki-67 staining. **K** Representative flow cytometry dot plots and histograms of Bcl-2 expression in thymic iNKT cells from WT and CD23 KO mice. **L** Quantification of the percentages of Bcl-2 positive cells and MFI showing significantly reduced Bcl-2 expression in CD23 KO mice (*n* = 6). Data are presented as mean ± SEM. Statistical significance was assessed using two-way ANOVA or a two-tailed unpaired Student’s *t* test. *p < 0.05, **p < 0.01, ***p < 0.001, ****p < 0.0001.

We next investigated whether CD23 deficiency affects apoptosis and proliferation of iNKT cells. In the thymus of CD23 KO mice, Annexin V^+^ and 7-AAD^+^ staining revealed a significantly increased frequency of apoptotic iNKT cells, whereas no differences were observed in the spleen compared with WT controls (Fig. 3A, B). Consistent with these findings, an increased proportion of Annexin V⁺7-AAD⁺ iNKT cells was also detected in the thymus of CD45.2 CD23 KO mice (Fig. 3A, C). Conversely, Ki-67 staining demonstrated that thymic iNKT cells from CD23-deficient mice displayed reduced proliferative capacity (Fig. 3D, E), a phenotype similarly evident in CD45.2 CD23 KO mice (Fig. 3D, F). In contrast, iNKT cell proliferation in the spleen of CD23 KO mice was indistinguishable from that in WT mice (Fig. 3D, E), with identical results observed in CD45.2 CD23 KO mice (Fig. 3D, F). To further confirm the proliferation and apoptosis of subsets of iNKT cells, we detected apoptosis of NKT1, NKT2, and NKT17 cells by Caspase-3 staining, which showed increased apoptosis of NKT17 cells in the thymus of CD23 KO mice (Fig. 3G, H). Reduced proliferation of NKT17 cells was observed in the thymus of CD23 KO and CD45.2 CD23 KO mice (Fig. 3I, J). T cell survival is mediated by the balance between intrinsic apoptotic pathways and the anti-apoptotic and pro-apoptotic members of the Bcl-2 family [28]. To delineate the mechanism underlying enhanced apoptosis in CD23-deficient iNKT cells, we assessed Bcl-2 expression. Both the frequency and expression levels of Bcl-2 were significantly reduced in thymic iNKT cells from CD23 KO mice (Fig. 3K, L), which is cell intrinsic as well (Supplementary Fig. S3E–H). By contrast, Bcl-2 expression in splenic iNKT cells from either CD23 KO or CD45.2 CD23 KO mice was indistinguishable from that in WT controls (Supplementary Fig. S3A–D, E–G, I). Collectively, these findings identify CD23 as a critical regulator of iNKT cell survival in the thymus, acting through the maintenance of Bcl-2 expression.

### CD23 is required for IL-7Rα-mediated iNKT cell differentiation

**Fig. 4:**
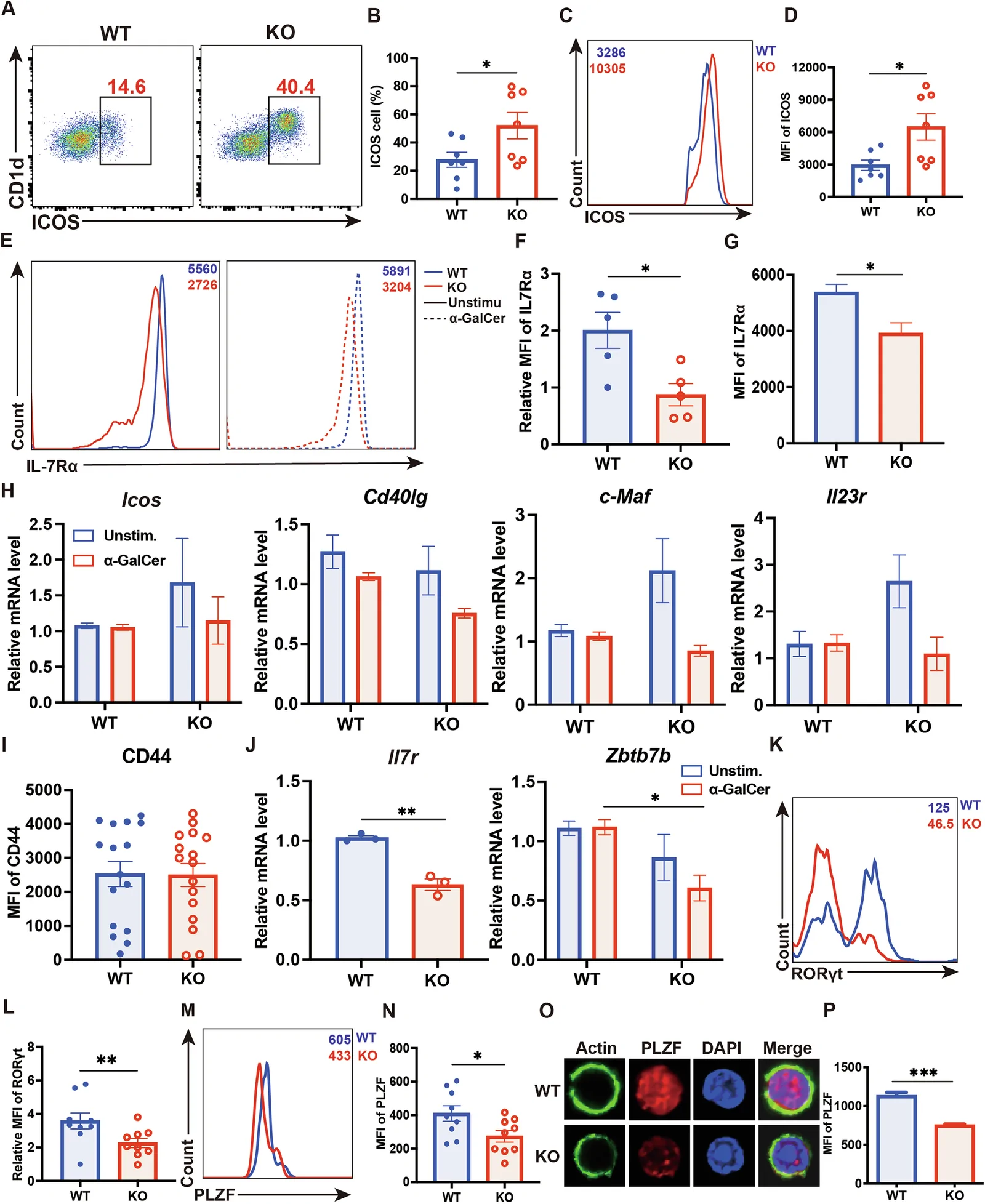
CD23 is required for IL-7Rα-mediated iNKT cell differentiation. **A** Representative flow cytometry analysis of ICOS expression on iNKT cells from WT and CD23 KO mice. **B** Percentage of ICOS-positive thymic iNKT cells from WT and CD23 KO mice (*n* = 7 per group). **C** Representative flow cytometry histogram overlays showing ICOS mean fluorescence intensity (MFI) on iNKT cells from WT and CD23 KO mice. **D** Quantification of ICOS MFI demonstrating significantly higher ICOS expression in CD23 KO mice (*n* = 7 per group). **E** Representative flow cytometry histograms comparing IL-7Rα expression on iNKT cells from WT and CD23 KO mice under unstimulated and α-GalCer-stimulated conditions. **F** Quantification of relative IL-7Rα MFI indicating a significant reduction in CD23 KO mice compared to WT mice (*n* = 5). **G** Quantification of IL-7Rα MFI levels after α-GalCer stimulation in WT and CD23 KO mice. **H** Relative mRNA expression levels of *Icos*, *Cd40lg*, *c-Maf*, and *Il23r* in unstimulated and α-GalCer-stimulated iNKT cells. **I** Mean fluorescence intensity (MFI) of CD44 on thymic iNKT cells from WT and CD23 KO mice (*n* = 16 per group). **J** Relative mRNA levels of *Il7r* and *Zbtb7b* in iNKT cells from WT and CD23 KO mice under unstimulated and α-GalCer-stimulated conditions. **K** Representative flow cytometry histogram overlays showing RORγt expression in WT and CD23 KO iNKT cells. **L** Quantification of relative RORγt MFI demonstrating a significant reduction in CD23 KO mice. **M** Representative flow cytometry histogram overlays showing PLZF expression in iNKT cells from WT and CD23 KO mice. **N** Quantification of PLZF MFI indicating significantly reduced levels in CD23 KO mice (*n* = 9 per group). **O** Representative immunofluorescence images of iNKT cells showing co-staining for PLZF, Actin, and DAPI in WT and CD23 KO mice. **P** Quantification of PLZF MFI from immunofluorescence staining confirming a significant reduction in CD23 KO mice. Data are presented as mean ± SEM. Statistical significance was assessed using two-way ANOVA or a two-tailed unpaired Student’s *t* test. *p < 0.05, **p < 0.01, ***p < 0.001.

Recent studies have demonstrated that the intensity of TCR signaling critically shapes iNKT effector subset development, with attenuated signaling favoring iNKT1 predominance, whereas stronger signaling promotes iNKT2 and iNKT17 differentiation [29, 30]. Co-stimulatory molecules provide essential secondary cues during iNKT cell maturation and effector polarization [31]. CD23-deficient iNKT cells exhibited an increased frequency of ICOS⁺ cells in the thymus (Fig. 4A, B), accompanied by markedly elevated ICOS expression levels (Fig. 4C, D). IL-7/IL-7R signaling is well established as a key regulator of iNKT and conventional T cell survival and homeostasis [23, 32]. Building on this, we assessed whether CD23 deficiency perturbs IL-7Rα expression and function in iNKT cells. Indeed, iNKT cells from CD23 KO mice exhibited significantly reduced IL-7Rα expression following α-GalCer stimulation compared with WT counterparts (Fig. 4E–G), suggesting that CD23 governs iNKT development through regulation of IL-7Rα. In parallel, we examined the expression of activation-associated genes and surface markers in iNKT cells. CD23 deficiency did not alter the mRNA levels of *Icos*, *Cd40lg*, *c-Maf*, or *Il23r*, nor the surface expression of CD44, compared with WT mice (Fig. 4H, I). In contrast, a marked reduction in *Il7r* and *Zbtb7b* transcript levels was observed in CD23-deficient iNKT cells (Fig. 4J). Notably, the lineage-specifying transcription factors RORγt and PLZF were markedly diminished in CD23-deficient iNKT cells (Fig. 4K–N). Consistent with these observations, intracellular immunofluorescence analysis revealed attenuated PLZF staining in KO cells (Fig. 4O, P), underscoring an essential role for CD23 in maintaining the differentiation program of iNKT and particularly NKT17 subsets. These data together reveal a critical role of CD23 in regulating IL-7Rα in the maintenance of iNKT cells.

### IL-7 regulates iNKT and NKT17 cell development and IL-17 production

**Fig. 5:**
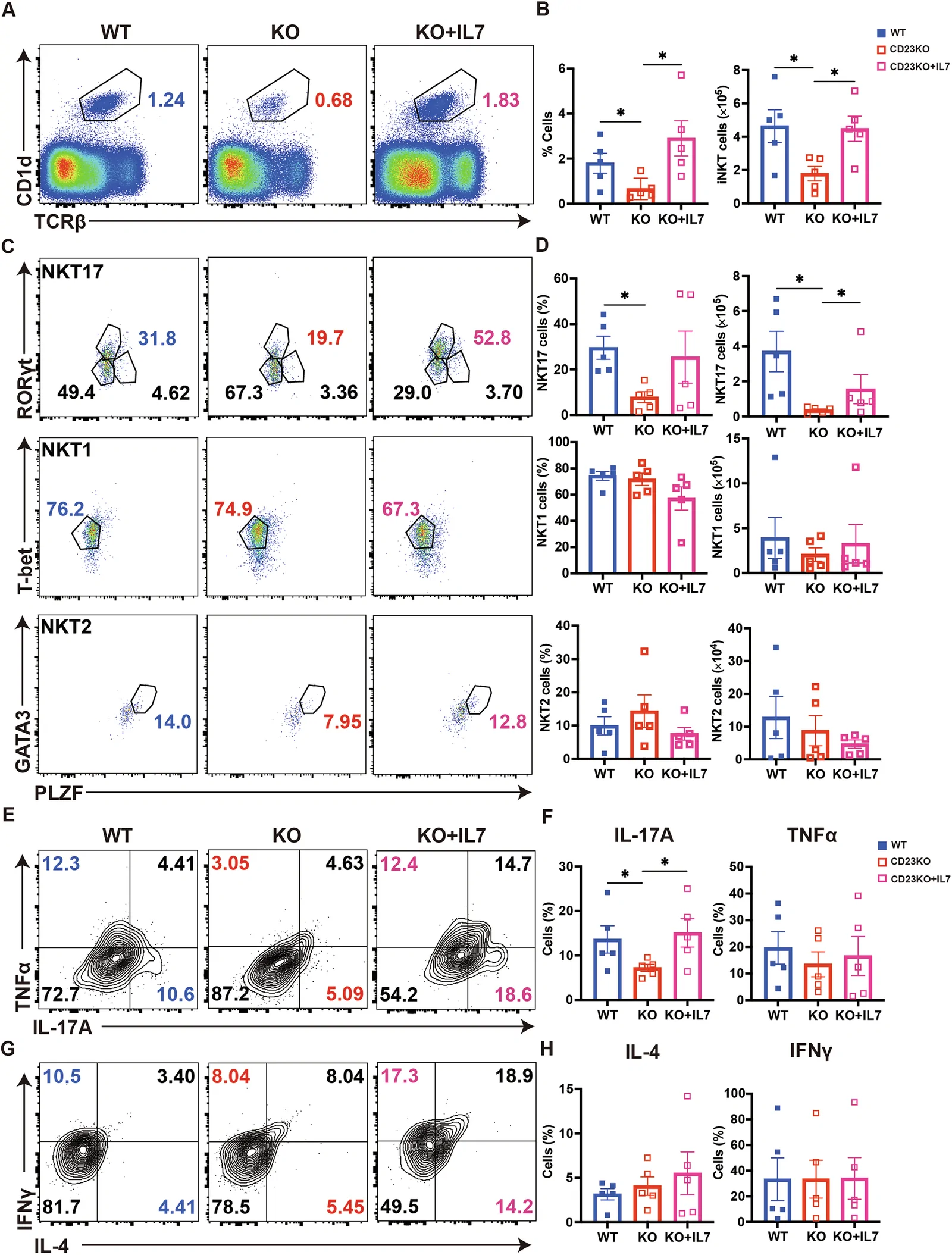
IL-7 regulates iNKT and NKT17 cell development and IL-17 production. **A**, **B** Representative flow cytometry plots and quantification of the percentages and absolute numbers of thymic iNKT cells in WT, CD23 KO, and in vivo IL-7-treated CD23 KO mice (*n* = 5 per group). **C**, **D** Representative flow cytometry plots and quantification of the percentages and absolute numbers of thymic NKT1, NKT2, and NKT17 cells in WT, CD23 KO, and in vivo IL-7-treated CD23 KO mice (*n* = 5 per group). **E**, **F** Representative flow cytometry plots and quantification of IL-17A and TNFα production by thymic iNKT cells from WT, CD23 KO, and in vivo IL-7-treated CD23 KO mice (*n* = 5 per group). **G**, **H** Representative flow cytometry plots and quantification of IL-4 and IFNγ production by thymic iNKT cells from WT, CD23 KO, and in vivo IL-7-treated CD23 KO mice (*n* = 5 per group). Data are presented as mean ± SEM. Statistical significance was assessed using one-way ANOVA. *p < 0.05.

To determine whether IL-7/IL-7Rα signaling plays a critical role in iNKT cell development, we administered IL-7 in vivo to CD23-deficient mice and assessed iNKT subset distribution. Flow cytometric analysis revealed that CD23 KO mice exhibited a marked reduction in both the frequency and absolute number of iNKT cells compared with WT controls. Notably, IL-7 administration significantly restored iNKT cell numbers in CD23 KO mice to levels comparable to WT counterparts (Fig. 5A, B). A similar trend was observed for the NKT17 subset: CD23 KO mice displayed a profound loss of NKT17 cells, which was largely rescued by IL-7 treatment (Fig. 5C, D). In contrast, IL-7 stimulation had little effect on the frequencies or absolute numbers of NKT1 and NKT2 cells (Fig. 5C, D). Cytokine profiling further demonstrated that CD23 KO mice had severely reduced IL-17A production relative to WT, which was efficiently restored by IL-7 treatment (Fig. 5E, F). By comparison, TNFα secretion remained unchanged across WT, CD23 KO, and IL-7-treated CD23 KO groups, and IL-7 stimulation failed to alter IL-4 or IFNγ production (Fig. 5E–H). These findings indicate that IL-7 selectively rescues iNKT and NKT17 cellularity and IL-17A secretion in the absence of CD23, underscoring a nonredundant role of IL-7 signaling in sustaining NKT17 homeostasis and effector function.

### CD23 regulates iNKT cell development through modulation of mTORC2 activation

**Fig. 6:**
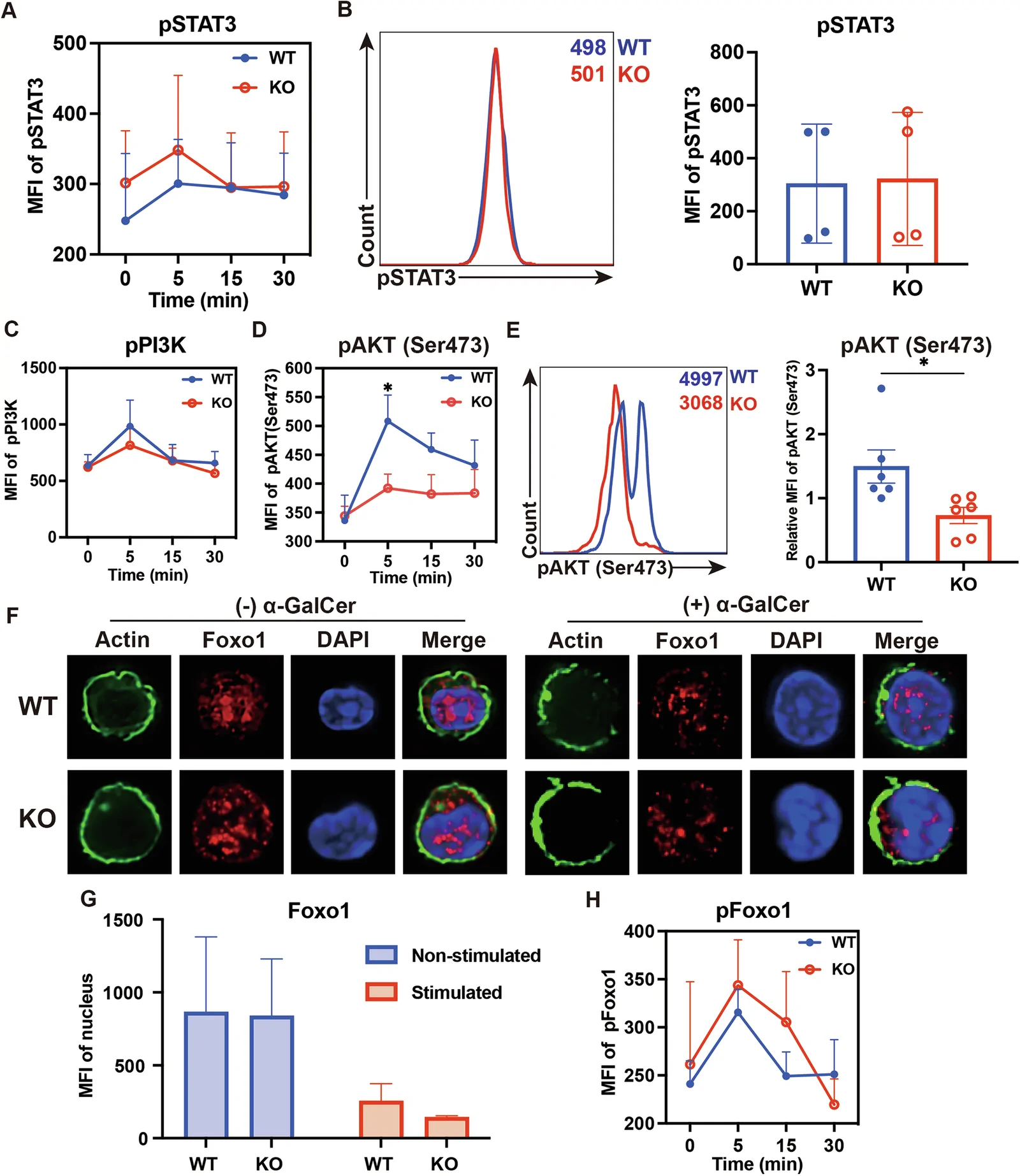
CD23 regulates iNKT cell development through modulation of mTORC2 activation. **A** Flow cytometry analysis of phosphorylated STAT3 (pSTAT3) mean fluorescence intensity (MFI) in iNKT cells from WT and CD23 KO mice (*n* = 3 per group) following stimulation with PMA plus ionomycin for 5 min, 15 min, and 30 min. **B** Representative flow cytometry histogram overlays (left) and quantification of pSTAT3 MFI (right) in WT and CD23 KO iNKT cells following α-GalCer stimulation in vitro (*n* = 4 per group). **C** Quantification of phosphorylated PI3K (pPI3K) MFI in iNKT cells from WT and CD23 KO mice (*n* = 5 per group) following stimulation with PMA plus ionomycin for 5 min, 15 min, and 30 min. **D** Quantification of phosphorylated AKT at Ser473 (pAKT (Ser473)) MFI in iNKT cells from WT and CD23 KO mice (*n* = 6 per group) following stimulation with PMA plus ionomycin for 5 min, 15 min, and 30 min. **E** Representative flow cytometry histogram overlays (left) and quantification of pAKT (Ser473) MFI (right) in iNKT cells from WT and CD23 KO mice following α-GalCer stimulation in vivo (*n* = 6 per group). **F** Representative immunofluorescence images of Foxo1 (red), Actin (green), and DAPI (blue) in iNKT cells under unstimulated and α-GalCer-stimulated conditions from WT and CD23 KO mice. **G** Quantification of nuclear Foxo1 MFI from immunofluorescence images, normalized to DAPI-defined nuclear area. No significant differences were observed between WT and CD23 KO mice under unstimulated or stimulated conditions. **H** Quantification of phosphorylated Foxo1 (pFoxo1) MFI in iNKT cells from WT and CD23 KO mice (*n* = 5 per group) following stimulation with PMA and ionomycin for 5 min, 15 min, and 30 min. Data are presented as mean ± SEM. Statistical significance was assessed using two-way ANOVA or a two-tailed unpaired Student’s *t* test. *p < 0.05.

STAT3 is a classical transcription factor essential for Th17 differentiation, acting via induction of RORγt and RORα to promote IL-17 expression [33]. However, previous studies have shown that IL-17⁺ iNKT cell development does not rely on IL-6/STAT3 signaling [34]. To assess whether CD23 deficiency affects iNKT cell development through altered STAT3 activation, we examined STAT3 phosphorylation following PMA and ionomycin stimulation. Phospho-flow analysis revealed comparable pSTAT3 levels between CD23 KO and WT iNKT cells (Fig. 6A). Likewise, α-GalCer stimulation did not alter pSTAT3 activation (Fig. 6B), suggesting that CD23 does not modulate the STAT3 pathway.

Given this, we next investigated whether CD23 regulates iNKT development via alternative signaling cascades known to control NKT17 differentiation, particularly the mTORC2 pathway. CD23 KO and WT iNKT cells exhibited similar pPI3K levels (Fig. 6C), indicating that upstream PI3K activation is unaffected. In contrast, phosphorylation of AKT at Ser473, a specific readout of mTORC2 activity [35], was markedly reduced in CD23-deficient iNKT cells, especially within 5 min after stimulation (Fig. 6D). α-GalCer activation further confirmed diminished pAKT (Ser473) in CD23 KO cells (Fig. 6E), indicating impaired mTORC2 signaling.

We further analyzed Foxo1, a transcriptional regulator downstream of AKT that exhibits subset-specific roles in iNKT cells. Foxo1 is critical for iNKT1 and iNKT2 differentiation but is largely dispensable for iNKT17 development [36]. In WT iNKT cells, strong nuclear localization of Foxo1 was observed under both basal and stimulated conditions, whereas CD23 KO iNKT cells displayed reduced nuclear Foxo1 staining (Fig. 6F, G). However, phosphorylation of Foxo1 was not significantly altered after stimulation (Fig. 6H), suggesting that CD23 loss does not substantially affect Foxo1 activity. Collectively, these data indicate that CD23 deficiency selectively impairs mTORC2 activation, as reflected by reduced AKT (Ser473) phosphorylation, thereby disrupting iNKT cell development and survival.

### IL-7 signaling sustains mTORC2 activity in iNKT cells and NKT17 cells

**Fig. 7:**
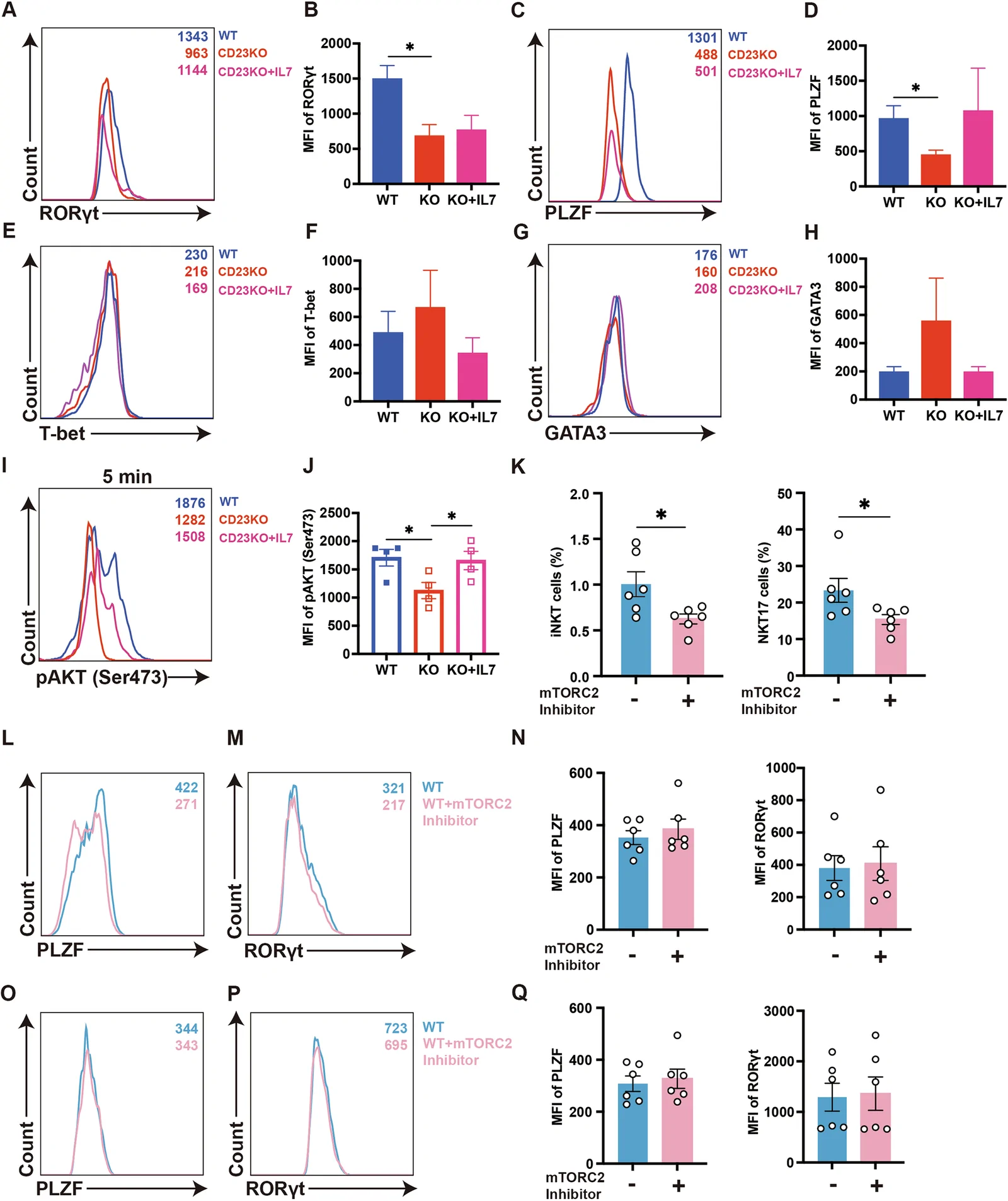
IL-7 signaling sustains mTORC2 activity in iNKT cells and NKT17 cells. **A** Representative flow cytometry histogram overlays showing RORγt expression levels in thymic iNKT cells from WT, CD23 KO, and in vivo IL-7-treated CD23 KO mice. **B** Quantification of the mean fluorescence intensity (MFI) of RORγt (*n* = 3 per group). **C** Representative flow cytometry histogram overlays showing PLZF expression levels in thymic iNKT cells from WT, CD23 KO, and in vivo IL-7-treated CD23 KO mice. **D** Quantification of PLZF MFI (*n* = 4 per group). **E** Representative flow cytometry histogram overlays showing T-bet expression levels in thymic iNKT cells from WT, CD23 KO, and in vivo IL-7-treated CD23 KO mice. **F** Quantification of T-bet MFI (*n* = 5 per group). **G** Representative flow cytometry histogram overlays showing GATA3 expression levels in thymic iNKT cells from WT, CD23 KO, and in vivo IL-7-treated CD23 KO mice. **H** Quantification of GATA3 MFI (*n* = 5 per group). **I** Representative flow cytometry histogram overlays showing phosphorylated AKT (Ser473) (pAKT (Ser473)) expression levels in thymic iNKT cells from WT, CD23 KO, and in vivo IL-7-treated CD23 KO mice following 5 min of PMA and ionomycin stimulation. **J** Quantification of pAKT (Ser473) MFI (*n* = 4 per group). **K** Proportions of iNKT and NKT17 cells in the thymus of BALB/c mice treated with or without an mTORC2 inhibitor JR-AB2-011 (*n* = 6 per group). Representative flow cytometry histogram overlays showing PLZF (**L**) and RORγt (**M**) expression levels in thymic iNKT cells from BALB/c mice treated with or without the mTORC2 inhibitor. **N** Quantification of PLZF and RORγt MFIs in thymic iNKT cells from BALB/c mice treated with or without the mTORC2 inhibitor (*n* = 6 per group). Representative flow cytometry histogram overlays showing PLZF (**O**) and RORγt (**P**) expression levels in thymic NKT17 cells from BALB/c mice treated with or without the mTORC2 inhibitor. **Q** Quantification of PLZF and RORγt MFIs in thymic NKT17 cells from BALB/c mice treated with or without the mTORC2 inhibitor (*n* = 6 per group). Data are presented as mean ± SEM. Statistical significance was assessed using one-way ANOVA or a two-tailed unpaired Student’s *t* test. *p < 0.05.

We next examined whether IL-7 could induce mTORC2 signaling in iNKT cells. The expression levels of the key transcription factors RORγt and PLZF were significantly reduced in CD23 KO mice compared with WT controls (Fig. 7A–D). However, in vivo administration of IL-7 did not significantly alter RORγt or PLZF expression in CD23 KO mice, suggesting that IL-7-mediated restoration of iNKT and NKT17 cells occurs independently of these transcription factors (Fig. 7A–D). Likewise, IL-7 stimulation did not affect the expression of T-bet or GATA3 in iNKT cells from CD23 KO mice (Fig. 7E–H). In contrast, the phosphorylation level of AKT at Ser473, a readout of mTORC2 activation, was markedly decreased in CD23 KO mice but was significantly restored upon IL-7 stimulation (Fig. 7I, J). These data indicate that IL-7 promotes the recovery of iNKT cells by activating the mTORC2 pathway, thereby enhancing AKT phosphorylation. To further confirm the role of mTORC2 in regulating iNKT and NKT17 cell homeostasis, WT mice were treated with a selective mTORC2 inhibitor. Inhibition of mTORC2 led to a pronounced reduction in the frequency of both iNKT and NKT17 cells (Fig. 7K), demonstrating the essential role of this pathway in maintaining these subsets. Notably, inhibition of mTORC2 did not significantly affect PLZF or RORγt expression in either total iNKT cells or the NKT17 subset (Fig. 7L–Q). Together, these findings demonstrate that IL-7/IL-7Rα signaling promotes iNKT cell development and NKT17 maintenance by activating the mTORC2-AKT pathway, independently of RORγt and PLZF expression.

## Discussion

This study identifies CD23 as a previously unrecognized regulator of iNKT cell development that operates through the IL-7Rα-mTORC2-AKT signaling pathway to selectively sustain NKT17 survival. Loss of CD23 leads to reduced IL-7Rα expression and impaired mTORC2-dependent phosphorylation of AKT (Ser473), accompanied by decreased Bcl-2 expression and enhanced apoptosis of NKT17 cells. Administration of exogenous IL-7 restores NKT17 frequency and IL-17 secretion in CD23-deficient mice without rescuing the reduced expression of PLZF and RORγt, suggesting that CD23 primarily supports survival and metabolic signaling rather than reprogramming lineage-defining transcription factors. Together, these findings position CD23 as an upstream checkpoint of the IL-7Rα-mTORC2 axis that safeguards NKT17 cell homeostasis.

Analysis of a publicly available scRNA-seq dataset of human bronchial brushings from allergic asthmatics (GSE193816) revealed a marked upregulation of *FCER2* (CD23) alongside altered iNKT cell signatures. Integrating this clinical observation with our mechanistic findings in mice, we hypothesize that elevated CD23 expression in the asthmatic airway may confer a survival advantage to local NKT17 cells. Specifically, upregulated CD23 may sustain IL-7Rα-mTORC2 signaling, driving the persistence and accumulation of these IL-17-producing innate-like T cells. Given the established role of IL-17 in driving airway hyperresponsiveness and neutrophilic infiltration [37], the CD23-mediated retention of NKT17 cells could exacerbate the inflammatory cascade. Consequently, the CD23-IL-7Rα-mTORC2 axis elucidated here not only governs the subset-specific homeostasis of iNKT cells, but also provides a mechanistic framework connecting elevated CD23 to human asthmatic pathology, nominating this axis as a potential therapeutic target for modulating IL-17-mediated airway inflammation.

Historically, CD23 has been studied as a low-affinity IgE receptor central to B cell biology and allergic responses [12, 15]. Earlier reports also noted CD23 expression on activated T cells [13], but its functional impact remained unclear. Our findings extend this paradigm by revealing a cell-intrinsic role of CD23 in iNKT cells, particularly in the NKT17 subset that is crucial for IL-17-mediated airway inflammation [9–11]. In line with this, CD23-deficient mice showed reduced airway hyperresponsiveness and inflammatory infiltration following α-GalCer challenge, implicating CD23 as a facilitator of IL-17-dependent airway pathology.

Mechanistically, these findings dovetail with and extend prior work implicating cytokine and metabolic pathways in iNKT subset differentiation. mTORC2 signaling has been established as a key determinant of iNKT cell fate. In particular, Rictor-dependent mTORC2 activity is essential for NKT17 development, as Rictor-deficient mice nearly lack thymic and peripheral NKT17 cells, whereas deletion of the PI3K inhibitor PTEN—thereby enhancing PI3K/Akt signaling—promotes NKT17 generation in a Rictor-dependent manner [20, 21]. Notably, Webster et al. demonstrated that the persistence and survival of IL-17-producing RORγt⁺ NKT17 cells are strictly dependent on IL-7 signaling, reflecting their intrinsically high expression of IL-7Rα [23]. Consistent with this, IL-7 can maintain NKT17 survival and proliferation through PI3K/Akt/mTOR signaling independently of TCR or STAT activation [23]. Our data align with and extend these observations, showing that CD23 deficiency reduces IL-7Rα expression and consequently impairs mTORC2-dependent AKT (Ser473) phosphorylation and Bcl-2 expression, leading to diminished NKT17 survival. Exogenous IL-7 restores pAKT (Ser473) levels and rescues NKT17 persistence even when RORγt and PLZF remain low, suggesting that CD23 primarily sustains IL-7-mediated metabolic and survival signaling rather than directly modulating lineage-defining transcription factors.

This survival-centered mechanism contrasts with classical transcriptional programming. PLZF directs the effector program of iNKT cells [38, 39], RORγt is indispensable for NKT17 fate [40], and GATA3 specifies NKT2 identity [41]. Other regulators highlight further layers: TSC1 constrains mTORC1 to prevent excessive NKT17 skewing [27], while Foxo1 promotes IL-7R expression and mTORC1 activity to sustain NKT1/2 but is dispensable for NKT17 [36]. These findings underscore a division of labor, in which transcription factors orchestrate lineage identity, whereas CD23-IL-7Rα-mTORC2 signaling ensures survival and persistence of NKT17 cells.

Additional pathways reinforce this paradigm. TSC1 constrains mTORC1 to prevent aberrant NKT17 skewing, with deficiency leading to elevated RORγt and ICOS expression [27]. Hippo/Mst signaling couples quiescence with maturation, with Mst1 deficiency impairing iNKT1 but favoring iNKT17 differentiation via altered mitochondrial dynamics [28]. Moreover, Raptor-dependent mTORC1 is indispensable for early maturation and NKT1 development, whereas Rictor/mTORC2 selectively supports NKT17 differentiation and IL-17 production [21]. Integrating these findings, our study positions CD23 as an upstream regulator that links IL-7Rα expression to mTORC2 activity, thereby safeguarding NKT17 survival without directly altering lineage transcriptional programs.

In conclusion, our work expands the immunological role of CD23 beyond IgE regulation, establishing it as a key checkpoint that sustains NKT17 survival through IL-7Rα-mTORC2-AKT signaling. From a translational perspective, these insights suggest that therapeutic modulation of the CD23-IL-7Rα-mTORC2 axis may offer opportunities for controlling iNKT/NKT17-driven airway inflammation. Nonetheless, limitations should be acknowledged: our findings are based on murine models, and direct validation in human iNKT/NKT17 cells is required.

Moreover, the precise molecular mechanisms by which CD23 regulates IL-7Rα expression remain unresolved. We offer the following speculative frameworks to guide future research. At the transcriptional level, since Foxo1 phosphorylation was unaltered in CD23-deficient iNKT cells, regulation likely proceeds via Foxo1-independent pathways (Fig. 6H). Given that the CD23 cytoplasmic domain recruits adaptor proteins and kinases such as Fyn [42, 43], analogous cascades could converge on *Il7r* regulators including GABPα or Ets-family members [44]. Post-transcriptionally, CD23 deficiency could alter metabolic cues that subsequently influence *Il7r* mRNA stability via specific microRNAs, such as miR-15/16, known to directly target this locus [45]. Most compellingly, given our observation that CD23 loss profoundly dampens IL-7Rα surface expression (Fig. 4E–J), a post-translational mechanism involving IL-7Rα surface stability warrants strong consideration. It has been established that IL-7Rα undergoes rapid clathrin-mediated internalization and JAK3-dependent degradation in T cells [46]. As a transmembrane glycoprotein capable of forming oligomeric surface clusters and undergoing dynamic recycling [42], CD23 might physically interact with IL-7Rα to facilitate its proper membrane localization or recycling, thereby shielding it from this premature degradation [46]. Future studies will be essential to validate these hypotheses and fully map the CD23-IL-7Rα regulatory interface.

Despite these caveats, delineating the CD23-IL-7Rα-mTORC2 axis provides a conceptual framework for targeted interventions, in which modulation of this pathway through recombinant cytokine therapy or small-molecule modulators could open new therapeutic avenues in asthma and other IL-17-mediated diseases.

## Materials and methods

### Mice

BALB/c and CD45.1 mice were purchased from GemPharmatech Co., Ltd. CD23^−/−^ mice were kindly provided by Prof. Chaohong Liu (Huazhong University of Science and Technology). Littermate controls and sex-matched mice (6–8 weeks old) were used for all experiments. Mice were maintained under specific pathogen-free conditions, and all procedures were approved by the Institutional Animal Care and Use Committee of the Children’s Hospital of Chongqing Medical University in accordance with institutional guidelines (Approval No. 2022-243).

### Bone marrow chimeric mice

Mixed bone marrow chimeras were generated by reconstituting sub-lethally irradiated (6 Gy) CD45.1^+^ recipient mice with a 1:1 mixture of bone marrow cells from CD45.2^+^ CD23 KO and CD45.1^+^ wild-type (WT) donors (5 × 10⁶ total cells/mouse). Immune reconstitution was analyzed 8 weeks post-transplantation.

### Flow cytometry analysis

Single-cell suspensions from thymus and spleen were stained with fluorochrome-conjugated antibodies in PBS containing 2% FBS. Surface markers: mCD1d-PBS57 tetramer (provided by the NIH Tetramer Facility), anti-TCRβ (H57-597), anti-CD4 (RM4-5), anti-CD8 (53-6.7), anti-CD19 (6D5), anti-CD23 (B3B4), anti-CD45.1 (A20), anti-CD45.2 (104), anti-Annexin V, anti-7-AAD, anti-ICOS (15F9), anti-IL7Rα (A7R34), anti-CD44 (IM7), and anti-IgG1 (M1-14D12). Intracellular molecules were analyzed using Foxp3 fixation/permeabilization buffers (Thermo Fisher Scientific, 00-5523-00) and the following antibodies: anti-Bcl-2 (BCL/10C4), anti-Ki-67 (16A8), anti-Caspase-3 (C92-605), anti-PLZF (D-9), anti-RORγt (AFKJS-9), anti-T-bet (4B10), anti-GATA3 (16E10A23), anti-TNFα (MP6-XT22), anti-IL-17A (TC11-18H10.1), anti-IFNγ (XMG1.2), and anti-IL-4 (11B11). All antibodies were purchased from BioLegend, BD Pharmingen, or Thermo Fisher Scientific. All samples were acquired on a FACS Celesta flow cytometer (BD Biosciences) and analyzed by FlowJo software (Tree Star).

### Phospho flow

Thymocytes were incubated with 7-AAD (BioLegend) and antibodies against CD44 (IM7, BioLegend), TCRβ (H57-597, BioLegend), CD4 (RM4-5, BioLegend), mCD1d-PBS57 tetramer, and CD8 (53-6.7, BioLegend) at 4 °C for 30 min. The thymocytes were then stimulated with PMA (50 ng/ml) and ionomycin (500 ng/ml) at 37 °C for 5, 15, and 30 min, followed by fixation with 4% paraformaldehyde, permeabilization, and staining for phosphorylated AKT (Ser473), STAT3 (Tyr705), PI3K (Tyr458/199), and Foxo1 (Ser256).

For α-GalCer stimulation, thymocytes (2 × 10⁶) were incubated with α-GalCer (125 ng/ml) for 72 h in a 24-well plate containing RPMI 1640 supplemented with 10% FBS. After stimulation, cells were fixed, permeabilized, and stained with PE-conjugated anti-phospho-STAT3 (pSTAT3, Tyr705) for 1 h. For AKT phosphorylation analysis following α-GalCer induction, mice were injected intraperitoneally with 5 μg of α-GalCer, and thymocytes were analyzed 2 h post-injection. Mean fluorescence intensity was recorded.

### Fluorescence microscopy

Sorted iNKT cells were cultured in RPMI 1640 supplemented with 10% FBS and either left unstimulated or stimulated with α-GalCer (125 ng/ml) for 72 h. After stimulation, cells were transferred onto poly-L-lysine-coated slides, incubated for 30 min, fixed with 4% paraformaldehyde, and permeabilized. For immunostaining, unstimulated cells were incubated with a mouse anti-PLZF antibody (4 μg/ml, Santa Cruz Biotechnology) and AF488-Actin (Invitrogen) for 1 h, followed by staining with a goat anti-mouse AF546 secondary antibody (1:400) for 30 min. Nuclei were counterstained with 1.5 μg/ml DAPI (Beyotime). Similarly, α-GalCer-stimulated cells were incubated with rabbit anti-Foxo1 (Cell Signaling Technology) and AF488-Actin for 1 h, followed by incubation with an AF546-conjugated goat anti-rabbit secondary antibody (1:400) for 30 min and counterstaining with DAPI. Images were acquired using a Nikon A1R confocal microscope.

### Airway hyperresponsiveness and inflammation

Airway hyperresponsiveness to methacholine was assessed 24 h after intranasal administration of 2 μg α-GalCer in 50 μl PBS. Mice were anesthetized, surgically fitted with a tracheal cannula, and placed on a ventilator (UGO BASILE S.R.L., Italy). Airway resistance was monitored using a pressure transducer. Bronchospasm was induced with nebulized methacholine (10, 25, 50, 100 mg/ml in 0.9% NaCl) delivered over 25 s at 130 breaths/min using a nebulization controller (Emka). Resistance was recorded at baseline and every 20 s for 5 min after each methacholine challenge, and values were averaged for each dose.

For histological analysis, lungs were fixed in formalin and stained with hematoxylin and eosin (H&E). Total RNA was extracted from lung tissue 5 h after α-GalCer injection using TRIzol reagent, and *Il17a* mRNA levels were quantified.

### Cytokine analysis

Thymocytes depleted of CD8^+^ cells were stimulated with PMA (50 ng/ml) and ionomycin (500 ng/ml) along with monensin (BD Pharmingen, 554724) for 5 h at 37 °C to assess cytokine production. For α-GalCer stimulation, thymocytes and splenocytes were stimulated for 72 h, with PMA and ionomycin along with monensin added during the final 5 h.

In vivo, mice were injected with 5 μg α-GalCer in 200 μl PBS, and thymocytes and splenocytes were isolated for intracellular cytokine analysis.

### RNA purification and real-time PCR

RNA was isolated using the RNeasy Micro Kit (QIAGEN) and cDNA was synthesized using the PrimeScript RT reagent Kit (TaKaRa). Real-time PCR was performed using SYBR Green Master Mix (QIAGEN) on a CFX96 Real-Time PCR system (Bio-Rad), with mouse *Gapdh* as an endogenous control. Relative gene expression was calculated using the 2^−ΔΔCT^ method. Primer sequences were: *Icos*, *c-Maf*, *Zbtb7b*, *Cd40lg*, *Il23r*, *Il17a*, and *Il7r*.

### Analysis of immune responses to α-GalCer

To assess in vivo responses, mice were injected intraperitoneally with 5 μg of α-GalCer in 200 μl PBS. After 2 h, thymocytes were isolated for IL-7Rα expression and pAKT (Ser473) levels in iNKT cells.

### In vivo administration

For IL-7 signaling analysis, IL-7 (1 μg) and InVivoMAb anti-mouse/human IL-7 (M25, Bio X Cell; 5 μg) were pre-incubated to form immune complexes. These complexes were injected intraperitoneally for 3 consecutive days. iNKT, NKT1, NKT2, NKT17 cells, and phosphorylated AKT (Ser473) were assessed.

To inhibit mTOR signaling in vivo, mice were injected daily with JR-AB2-011 (20 mg/kg) for 5 days. The control group received an equal volume of the corresponding vehicle.

### Statistical analysis

Sample sizes were determined based on prior literature and preliminary experiments to ensure adequate power to detect biologically meaningful differences; no formal statistical method was used to predetermine sample size. No samples or animals were excluded from the analysis, and no predefined inclusion or exclusion criteria were applied. Animals were randomly assigned to experimental groups for all in vivo treatments. Investigators were not blinded to group allocation during the experiments or outcome assessment. All data are displayed as mean ± SEM. *P* values were calculated with two-tailed unpaired Student’s *t* test, one-way ANOVA, and two-way ANOVA as indicated in the figure legends (GraphPad Prism). Statistical significance was as follows: *p < 0.05; **p < 0.01; ***p < 0.001; ****p < 0.0001.

## Supplementary information

Supplementary Materials for CD23 sustains NKT17 cell homeostasis via IL-7Rα–mTORC2 signaling

Supplementary Table S1 for CD23 sustains NKT17 cell homeostasis via IL-7Rα–mTORC2 signaling

## Data Availability

All data supporting the findings of this study are available within the article and its Supplementary Information files. Additional data are available from the corresponding author upon reasonable request. The scRNA-seq dataset analyzed in this study was obtained from the Gene Expression Omnibus (GEO) under accession number GSE193816.

## Code availability

No custom or proprietary mathematical algorithms were used in this study. All bioinformatics and statistical analyses were performed using R software (version 4.5.0). Single-cell RNA sequencing data processing, dimensionality reduction, and cell clustering were conducted using the Seurat (version 5.5.0), SingleCellExperiment (version 1.30.1), and zellkonverter (version 1.18.0) packages. Gene signature scoring and functional enrichment analyses were performed using GSVA (version 2.2.1). Data manipulation and statistical testing were supported by the tidyverse (version 2.0.0), rstatix (version 0.7.3), and Hmisc (version 5.2.5) packages. Data visualization was implemented using ggplot2 (version 4.0.3), ggpubr (version 0.6.3). Analysis scripts used to generate the figures and results are available from the corresponding author upon reasonable request.
