# Supplementary material for: CD23 sustains NKT17 cell homeostasis via IL-7Rα-mTORC2 signaling
Source: Cell Death Dis. 2026 Jun 30;17(1):806. doi: 10.1038/s41419-026-09060-x (PMC13586390; doi:10.1038/s41419-026-09060-x)
Supplement: Supplementary file 1 [file 41419_2026_9060_MOESM1_ESM.docx]

Supplementary Materials for

**CD23 sustains NKT17 cell homeostasis via IL-7Rα-mTORC2 signaling**

Linlin Niu^1†^, Bingqian Tan ^1†^, Jingting Xu ^1, 2^, Guangwei Zhang^1^, Bo Wang^1^, Zuochen Du^1, 3, 4^, Di Yang^1, 3^, Chengxi Zhuang^1^, Xiaoyu Du^1^, Yao Zhao^1^*

Corresponding author: [Zhaoy@cqmu.edu.cn](mailto:Zhaoy@cqmu.edu.cn)

**Fig. S1** S**ingle-cell transcriptome analysis reveals immune cell lineage alterations in allergic asthma. (A) t-SNE visualization of overall immune cell distribution in allergic asthmatics (AA) and allergic controls (AC) (n = 4 for AA; n = 4 for AC). (B) Clustering and annotation of major subpopulations, including CD4^+^ and CD8^+^ T cells, iNKT cells, and MAIT cells. (C) Quantitative analysis of inter-individual variability in specific T cell subsets between AA and AC. (D) Comparison of immune cell signature scores in CD4^+^ naive T cells, CD4^+^ memory T, CD8^+^ T cells, iNKT cells, Tregs, naive and memory B cells, activated NK cells, and M1/M2 macrophages. (E, F) Gene expression analysis of *CD81*, *CR2*, *CD19*, *FCER1G*, *FCER2* (CD23), and *FCER1A*.**

**Fig. S2 CD23 deficiency selectively impairs NKT17 cell development and IL-17 production in iNKT cells.** (A) Representative flow cytometry plots showing thymic and splenic iNKT subsets (NKT1, NKT2, and NKT17) from bone marrow chimeric mice reconstituted with CD45.2 WT or CD45.2 CD23 KO cells. Cells were gated on CD1d-PBS57⁺TCRβ⁺ iNKT populations and analyzed for PLZF, RORγt, T-bet, and GATA3 expression. (B, C) Quantification of the frequencies and absolute numbers of NKT1, NKT2, and NKT17 subsets in the thymus (B) and spleen (C) from CD45.2 WT or CD45.2 CD23 KO mice (n = 6 per group). (D-F) Flow cytometric analysis of cytokine production by iNKT cells following *in vitro* α-GalCer stimulation for 3 days. Representative flow cytometry plots and bar graphs showing intracellular staining of IL-17A, TNFα, IFNγ, and IL-4 in thymic (Thy) and splenic (Spl) iNKT cells from WT and CD23 KO mice (n = 6 per group). (G-I) Representative flow cytometry plots and quantification of IL-17A, TNFα, IFNγ, and IL-4 production by thymic (Thy) (n = 4) and splenic (Spl) (n = 3) iNKT cells from WT and CD23 KO mice following *in vivo* α-GalCer challenge. (J-L) Flow cytometric analysis of thymic iNKT cells from CD45.2 WT and CD45.2 CD23 KO mice (n =6 per group) showing a reduction of IL-17⁺ iNKT cells. Data are presented as mean ± SEM. Statistical significance was assessed using two-way ANOVA. **p* < 0.05, ***p* < 0.01.

**Fig. S3 Analysis of Bcl-2 expression in iNKT cells from CD23-deficient and WT mice.** (A) Representative flow cytometry plots showing Bcl-2 expression in splenic iNKT cells from WT and CD23 KO mice. (B) The percentages of Bcl-2⁺ iNKT cells in the spleen from WT and CD23 KO mice (n = 7 per group). (C, D) Representative flow cytometry histogram overlays and quantification of Bcl-2 MFI in splenic iNKT cells from WT and CD23 KO mice (n = 7 per group). (E) Representative flow cytometry plots showing Bcl-2 expression in thymic and splenic iNKT cells from CD45.2 WT and CD45.2 CD23 KO mice. (F) Frequencies of Bcl-2-positive iNKT cells in the thymus and spleen from CD45.2 WT and CD45.2 CD23 KO mice (n = 6 per group). (G-I) Representative flow cytometry histogram overlays and quantification of Bcl-2 MFI in thymic and splenic iNKT cells from CD45.2 WT and CD45.2 CD23 KO mice (n = 6 per group). Data are presented as mean ± SEM. Statistical significance was assessed using a two-tailed unpaired Student’s t test. ***p* < 0.01, ****p* < 0.001.
